# Relationship Between Hepatitis C Clinical Testing Site and Linkage to Care

**DOI:** 10.1093/ofid/ofu009

**Published:** 2014-05-14

**Authors:** Sabrina A. Assoumou, Wei Huang, C. Robert Horsburgh, Mari-Lynn Drainoni, Benjamin P. Linas

**Affiliations:** 1 Department of Medicine, Section of Infectious Diseases, Boston University School of Medicine, Boston, Massachusetts; 2 Department of Health Policy and Management; 3 Department of Epidemiology, Boston University School of Public Health, Boston Massachusetts; 4 Center for Healthcare Organization and Implementation Research, Boston, Massachusetts

**Keywords:** Diagnosis, hepatitis C, outcome

## Abstract

**Background.:**

The Centers for Disease Control and Prevention recommends one-time hepatitis C virus (HCV) testing of the population born between 1945 and 1965 with follow-up RNA testing for those with reactive serology. To increase the rate of diagnosis, testing may be considered in settings other than outpatient clinics (OC), such as inpatient wards (IP) or emergency department (ED).

**Methods.:**

We used electronic medical records to create a retrospective cohort with reactive HCV serology between 2005 and 2010 at an urban safety net hospital. We determined factors associated with linkage to HCV care as measured by HCV RNA testing, and we evaluated the rate of linkage to care according to diagnosis location (OC, IP, or ED).

**Results.:**

Individuals, 37 828, were tested and 5885 (16%) were reactive. Seropositivity was similar across all sites. Of the 4466 patients who met inclusion criteria, 3400 (76%) were diagnosed in the OC, whereas 967 (22%) and 99 (2%) were tested in the IP and the ED, respectively. A total of 2135 (48%) underwent HCV RNA testing. Using multivariable regression modeling, the following factors were independently associated with HCV RNA testing: diagnosis in the OC (odds ratio [OR], 1.64; 95% confidence interval [CI], 1.42–1.90); age at diagnosis in decades (OR, 0.98; 95% CI, 0.98–0.99); private insurance (OR, 1.17; 95% CI, 1.01–1.34); and ≥10 visits after diagnosis (OR, 2.15; 95% CI, 1.89–2.44).

**Conclusion.:**

There is an opportunity to increase HCV diagnosis by testing in sites other than the OC, but this opportunity needs to be coupled with robust initiatives to improve linkage to care.

## Abstract (precis)

When compared to those diagnosed in the outpatient, patients with reactive HCV testing in the Emergency Department or in the inpatient setting were less likely to link to care as measured by HCV RNA testing.

Hepatitis C virus (HCV) is an important public health problem that affects 3.2 million people in the United States [1, 2]. For the first time, the nation's 10-year public health objectives—collectively known as “Healthy People 2020”—include HCV-specific goals such as reducing the rate of new HCV infections and increasing the proportion of individuals aware of their HCV diagnosis [3]. In addition, the Centers for Disease Control and Prevention (CDC) and the United States Preventive Services Task Force (USPSTF) have been recommending one-time HCV testing for persons born between 1945 and 1965 [4, 5] since 2012 and 2013, respectively. Currently, testing for HCV infection in outpatient clinics is infrequent [6]. Many persons at risk for HCV infection have histories of current or past injection drug use, and they do not have access to routine healthcare through a primary care provider [7]. To increase HCV case identification, it will likely be necessary to test for HCV in healthcare settings other than outpatient clinics (OC), including inpatient wards (IP) or emergency departments (ED), where testing for other infectious diseases with similar risk factors, such as human immunodeficiency virus (HIV), has already been implemented (8). Understanding current rates of HCV diagnosis in the OC, IP, and ED, as well as the extent to which patients identified in each environment ultimately link to and initiate HCV-related care, is critical information for developing future testing efforts.

We used the electronic medical record of Boston Medical Center (BMC), a 500-bed urban safety net hospital with a high prevalence of HCV [9], to: (1) determine the frequency of HCV diagnosis in the OC, IP, and ED settings within this safety net hospital; (2) understand the demographics of patients diagnosed in each clinical setting; (3) and investigate the extent to which HCV-infected individuals identified in each clinical setting ultimately link to HCV care as measured by HCV RNA testing according to CDC guidelines.

## MATERIALS AND METHODS

### Overview

We used BMC's electronic medical record to create a retrospective cohort of patients who had reactive HCV serology between 2005 and 2010, and we used standard statistical methods to investigate HCV outcomes stratified by site of diagnosis. The 3 sites considered were (1) the IP, (2) OC, and (3) the ED. Examples of OC included primary care or subspecialist clinics.

### Site

Boston Medical Center is a safety net hospital caring for an underserved community. Approximately two-thirds of the patient population are persons from racial or ethnic minority groups and approximately 70% come from underserved populations, including low-income families and immigrants [10].

### Primary and Secondary Outcomes

The primary outcome, HCV RNA testing, was used as a proxy for initiation of HCV-related care and linkage to care in line with the new CDC guidance recommending follow-up of any reactive HCV antibody with HCV RNA testing [11]. We also wanted to describe the population diagnosed with HCV at BMC stratified by different testing sites and to determine factors associated with linkage to care. We also performed a sensitivity analysis by restricting the evaluation to the patients who were specifically listed as having BMC as the location of their primary care provider. This process was done to determine whether results were biased by including individuals whose primary care providers were outside the system.

### Study Population

Inclusion criteria were as follows: (1) reactive HCV antibody; (2) diagnosis between January 1, 2005 and December 31, 2010; (3) at least 12 months of follow-up time after initial reactive HCV serology.

### Data Collection

Data elements included demographic information, laboratory values, and dates and locations of all clinical visits.

### Independent Variables

Covariates included in the analyses were age at baseline, gender, race or ethnicity, insurance type (private vs public), birthplace (United States vs foreign born), number of follow-up visits, and diagnosis location (OC vs IP or ED).

### Statistical Analyses

We used descriptive statistics to determine the proportion of patients identified in OC, IP, and ED. We then calculated the proportion of patients with each outcome of interest (eg, testing for HCV RNA vs no testing for HCV RNA). Logistic regression was used to evaluate predictors of HCV-related care completion. Variables significant in univariate analysis and confounders were included in a multivariable model. We calculated odds ratios (ORs) of receiving HCV RNA, as well as 95% confidence intervals (CIs). All *P* value significance levels were two-sided. Statistical analyses were performed with STATA 12 (STATA, College Station, TX).

### Ethics

The Boston University Medical Center Institutional Review Board approved this study.

## RESULTS

**Figure 1.**
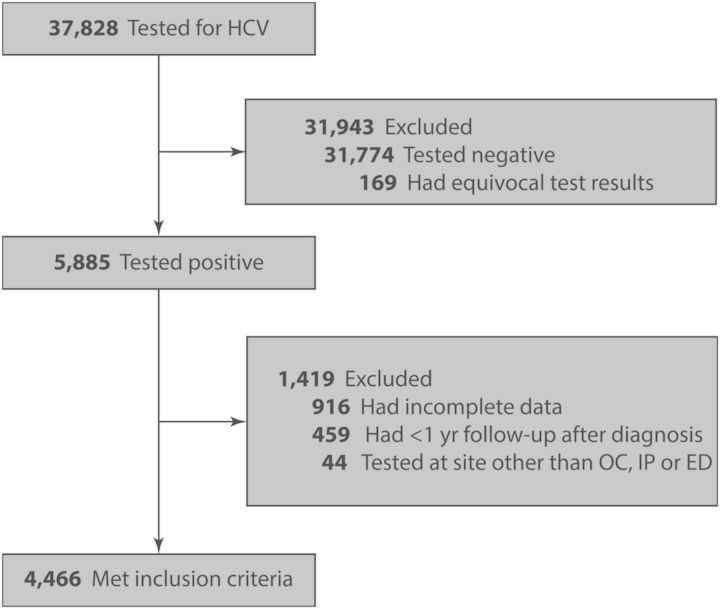
Study flow diagram. Abbreviations: ED, emergency department; HCV, hepatitis C virus; IP, inpatient wards; OC, outpatient clinics.

We identified 37 828 unique patients who underwent HCV testing; of those 5885 (16%) were reactive. A total of 4466 individuals met inclusion criteria after we excluded the following patients: 44 who were tested in a site other than the OC, IP, or ED; 459 who did not have at least 1 year of follow-up time after diagnosis; and 916 who had incomplete information (Figure 1). Of the 4466 patients meeting inclusion criteria, 3400 (76%) were diagnosed in the OC, whereas 967 (22%) and 99 (2%) were tested in the IP and the ED, respectively (Table 1). There was a median of 11 months of follow-up time (range, 0.4–81 months), and the median number of follow-up visits after diagnosis was 14. The cohort was 65% male; 45% were White, 32% Black, 19% Latino, 3% Asian, and 2% other or unknown. Eight hundred eighty-five (20%) were foreign born and 2885 (65%) were covered by public insurance. There were 431 (10%) patients infected with HIV.

**Table 1.** Baseline Characteristics

Characteristics	Total N = 4466 N (%)	Outpatient N = 3400 N (%)	Inpatient N = 967 N (%)	Emergency N = 99 N (%)	*P* Value
Age at HCV diagnosis					
Mean (SD)	44	44	45	39	<.001
Median	46	46	47	40	
Range	18–89	18–89	18–88	18–77	
Age groups					
18–39	1443 (32)	1090 (32)	305 (32)	48 (48)	<.001
40–69	2917 (65)	2241 (66)	627 (65)	49 (49)	
≥70	106 (3)	69 (2)	35 (4)	2 (2)	
Male	2911 (65)	2261 (67)	594 (61)	56 (57)	.003
Race/Ethnicity					
White	1994 (45)	1500 (44)	456 (47)	38 (38)	.23
Black	1410 (32)	1068 (31)	308 (32)	34 (34)	
Latino	829 (19)	644 (19)	164 (17)	21 (21)	
Asian	129 (3)	104 (3)	20 (2)	5 (5)	
Other/Unknown	104 (2)	84 (2)	19 (2)	1 (1)	
History of HIV infection	431 (10)	365 (11)	60 (6)	6 (6)	<.001
Insurance					
Public	2885 (65)	2103 (62)	712 (74)	70 (71)	<.001
Private	1159 (26)	952 (28)	189 (20)	18 (18)	
Other/Unknown	422 (9)	345 (10)	66 (7)	11 (11)	
Birthplace					
US	3415 (76)	2579 (76)	761 (79)	75 (76)	0.054
Non-US	885 (20)	698 (21)	164 (17)	23 (23)	
Unknown	166 (4)	123 (4)	42 (4)	1 (1)	
No. of visits after diagnosis					
Mean	25	25	22	23	.01
Median	14	15	12	11	
Range	1–258	1–258	1–221	1–194	
Follow-up time, months					
Mean	18	17	17	24	<.001
Median	11	11	11	18	
Range	(0.4–81)	(1–81)	(0.4–79)	(1–79)	

Abbreviations: HCV, hepatitis C virus; HIV, human immunodeficiency virus; SD, standard deviation; US, United States.

**Figure 2.**
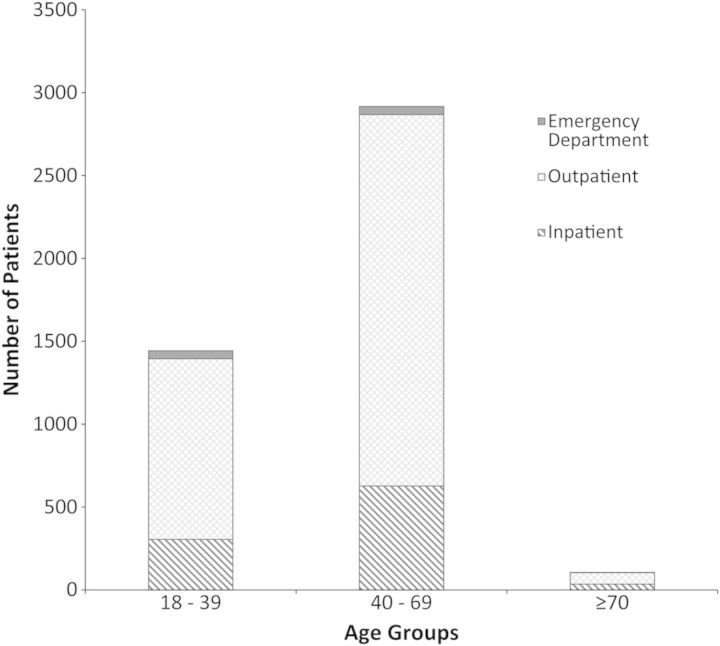
Hepatitis C-infected patients stratified by age groups and diagnosis location. Abbreviations: ED, emergency department; IP, inpatient wards; OC, outpatient clinics.

Figure 2 stratifies the cohort by age groups (ages 18–39, 40–69, and ≥70). The majority of patients with reactive HCV serology were in the 40–69 age group, and testing was performed in the outpatient. Hepatitis C seropositivity rates were similar across the 3 settings evaluated. In the OC, 11% of individuals tested were seropositive. Likewise, 14% and 12% of individuals tested were HCV-infected in IP and ED, respectively.

### Primary Outcome

Of the 4466 patients meeting inclusion criteria, 2135 (48%) underwent HCV RNA testing and 245 (5%) initiated treatment (Table 2). The ED and IP populations were similar with regard to risk for HCV RNA testing, and therefore these groups were combined in the multivariate model. After multivariable modeling controlling for diagnosis location (OC vs IP or ED), age at diagnosis (in decades), gender, race, insurance type, birthplace, and number of follow-up visits, the following factors were independently associated with HCV RNA testing: diagnosis in the OC (OR, 1.64; 95% CI, 1.42–1.90); age at HCV diagnosis in decades (OR, 0.98; 95% CI, 0.98–0.99); private insurance (OR, 1.17; 95% CI, 1.01–1.34); and ≥10 visits after HCV diagnosis (OR, 2.15; 95% CI, 1.89–2.44) (Table 3). Although the number of follow-up visits was significantly associated with a higher likelihood of receiving HCV RNA testing among those who had at least 10 follow-up visits, only 48% received HCV RNA testing. We also performed an analysis by only including the 1910 patients who were specifically listed as having BMC as the location of their primary care provider. We found results similar to the main analysis. Hepatitis C virus RNA testing was significantly associated with the following: diagnosis in the OC, age at diagnosis (in decades), male gender, Asian race or ethnicity, and ≥10 clinical visits after diagnosis (Supplementary Table 1).

**Table 2.** Hepatitis C General and Treatment-Related Care

Characteristics	Total (N = 4466) N (%)	Outpatient (N = 3467) N (%)	Inpatient (N = 988) N (%)	Emergency (N = 99) N (%)
HCV treatment-related care				
HCV RNA testing	2135 (48)	1734 (51)	362 (37)	39 (39)
Genotyping	672 (15)	574 (17)	88 (9)	10 (10)
Treatment	245 (5)	218 (6)	25 (3)	2 (2)
HCV general care				
Hepatitis A vaccination	449 (10)	397 (12)	48 (5)	4 (4)
Hepatitis B vaccination	1174 (26)	990 (29)	160 (17)	24 (24)

Abbreviation: HCV, hepatitis C virus.

**Table 3.** Factors Associated With Hepatitis C RNA Testing (N = 4466)*

Predictors	Univariate Odds Ratio (95% CI)	Univariate *P* Value	Adjusted Odds Ratio (95% CI)	Adjusted *P* Value
Age at HCV diagnosis	0.99 (0.98–0.99)	<.001	0.98 (0.98–0.99)	<.001
Male	0.99 (0.87–1.12)	.82	1.07 (0.94–1.22)	.29
Race/Ethnicity				
White	Ref.		Ref.	
Black	1.20 (0.84–1.72)	.31	1.11 (0.74–1.65)	.64
Latino	0.84 (0.73–0.97)	.01	0.87 (0.75–1.01)	.07
Asian	1.05 (0.89–1.23)	.57	0.88 (0.71–1.09)	.25
Other/Unknown	0.86 (0.58–1.28)	.46	0.88 (0.57–1.36)	.57
Insurance				
Public	Ref.		Ref.	
Private	1.24 (1.08–1.42)	.002	1.17 (1.01–1.34)	.03
Other/Unknown	1.07 (0.87–1.31)	.54	1.13 (0.91–1.40)	.26
Birthplace				
US	Ref.		Ref.	
Non-US	1.18 (1.01–1.36)	.03	1.27 (1.03–1.57)	.03
Unknown	0.78 (0.56–1.06)	.12	1.01 (0.72–1.41)	.95
Diagnosis location				
IP/ED	Ref.		Ref.	
Outpatient	1.73 (1.50–1.99)	<.001	1.64 (1.42–1.90)	<.001
Clinical visits after diagnosis				
<10	Ref.		Ref.	
≥10	2.03 (1.79–2.29)	<.001	2.15 (1.89–2.44)	<.001

Abbreviations: CI, confidence interval; ED, emergency department; HCV, hepatitis C virus; HIV, human immunodeficiency virus; IP, inpatient wards; IQR, interquartile range; Ref., Reference group; US, United States.

*This analysis controls for age (in decades), gender, ethnicity, insurance type, birthplace, the diagnosis location, and the number of clinical visits.

## DISCUSSION

More effective HCV therapy has increased the enthusiasm for identifying and treating HCV, and the CDC and USPSTF have expanded their guidelines to include one-time testing of the cohort born between 1945 and 1965. The juxtaposition of these factors, combined with the high proportion of HCV-infected unaware of their diagnosis, likely indicates that large numbers of persons will be newly diagnosed in the coming years. Because public health initiatives are committed to increase the rate of HCV testing, it is important to understand where testing is currently occurring. In addition, stratifying outcomes by diagnosis location is essential to design an effective program to expand access to HCV care. Our data show that the majority (76%) of HCV testing is occurring in the OC and that individuals diagnosed in the OC are more likely to link to HCV care. In addition, we found that HCV seropositivity rates were similar across the 3 settings evaluated.

Because little HCV testing is occurring in IP and ED, there seems to be an opportunity for expanding testing in those 2 locations. Nevertheless, the usefulness of testing in those settings is currently limited by findings that approximately 60% of patients diagnosed in the IP and ED did not link to HCV care. Our data suggest that if testing were to be increased in those 2 settings, it would need to be reinforced by services to improve linkage to HCV care. The ED could be a particularly important setting where those with limited access to health services could be reached. Studies have shown that it is often the entryway to the healthcare system for many patients with low income [12]. We also found that there was a median of 14 visits in our system after HCV diagnosis. This result suggests that there might have been missed opportunities to initiate HCV-related care. Given the projected burden of HCV disease if patients were to remain unaware of their diagnosis, there should be an effort to use any interactions with the health system as an opportunity for testing.

Our seropositivity of 16% is similar to other studies carried out in urban safety net hospitals. For example, a retrospective study performed at Metrohealth in Cleveland reported a 13% HCV seropositivity [13]. Such high proportions suggest that increasing testing rates at urban safety net hospitals could increase the number of patients who are aware of their HCV status in the United States. We also found that 52% of patients did not have HCV RNA testing, which is in line with prior studies. An analysis utilizing 2006–2007 surveillance data in 6 US locations revealed that 46% of patients with reactive HCV antibody did not have HCV RNA testing [14]. Another prospective cohort study of 8810 patients engaged in HCV care between 2006 and 2008 at 4 integrated healthcare systems in the United States reported that 37% were never tested for HCV RNA [15].

There are several limitations to our study. The retrospective study design and the use of single-site data may limit generalizability. Furthermore, patients who did not follow-up at BMC may have received HCV-related care elsewhere. We attempted to address this issue by performing a sensitivity analysis in which we restricted our evaluation to patients who listed BMC as their primary care site. This analysis yielded very similar findings. Another limitation of the study is that we were not able to control for factors such as active injection drug use or mental illness. We relied on International Classification of Diseases (ICD)-9 codes for comorbidities, and a review of the data showed that these factors were not well documented, especially in certain testing sites such as the ED or the IP. It is possible that some sites might not perform as well because of unmeasured confounders. Because this is a retrospective study, we were not able to address causality. We noted that linkage to care was lower in certain sites, and this observation needs to be taken into account if more widespread testing were to be carried out in the ED or the IP. We cannot conclude that testing in the ED or IP leads to less HCV RNA testing, but we are observing that those diagnosed in the ED or IP are less likely to have this particular testing performed. Future research is needed to determine the reasons for our observation. There are also limitations associated with using HCV RNA testing as a proxy for linkage to care. Although this test is the first step in initiating HCV-related care, HCV RNA testing does not necessarily indicate that patients were evaluated by a provider qualified to address HCV-related care. In addition, a visit with a qualified provider does not necessarily indicate initiation of HCV-related care. Furthermore, our observed linkage to care rate remains low even when one considers that HCV RNA testing might overestimate linkage to care because some sites might automatically reflex to RNA testing when HCV antibody is identified.

In addition, we did not have information on the reasons for ordering HCV tests. It is possible that reasons for testing in the ED and the IP setting might have been different than in the OC, and this characteristic might have influenced downstream documentation of HCV-related care. For example, the ED might have a high rate of testing of occupational and nonoccupational exposures to blood-borne pathogens, and those particular cases might be more likely to see out-of-network providers. Follow-up testing performed by those physicians might not have been available in our medical records.

In conclusion, our study showed that at a large urban safety net hospital, HCV seropositivity rates were similar across the 3 clinical settings evaluated. Forty-eight percent of patients diagnosed with HCV received subsequent HCV-related care in the form of HCV RNA testing. Individuals tested either in the IP or in the ED were less likely to link to HCV care when compared to the outpatient clinical setting. As HCV testing is expanded in light of new guidelines, the IP and the ED are potential locations to evaluate a difficult-to-reach population; however, testing needs to be combined with interventions to ensure that those diagnosed with HCV in these nontraditional settings are evaluated for and receive subsequent HCV-related care.
